# The First Chew

**Published:** 1890-11

**Authors:** 


### THE FIRST CHEW.

The boy said it was a peculiar kind of tobacco, and was known as molasses tobacco because it was so sweet. The other boys did not ask how he came to know its name, or where he got it—boys never ask any thing that it would be well for them to know—but they accepted his theory and his further statement that it was of a mildness singularly adapted to learners without misgiving. The boy was himself chewing vigorously on a large quid, and launching the juice from his lips right and left like a grown person, and my boy took as large a bit as his benefactor bade him. He found it as sweet as he had been told it was, and he acknowledged the aptness of its name of molasses tobacco. It seemed to him a golden opportunity to acquire a noble habit on easy terms. He let the quid rest in his cheek, as he had seen men do, when he was not crushing it between his teeth, and for some moments he poled his plank up and down the canal boat with a sense of triumph that nothing marred.

Then all of a sudden he began to feel pale. The boat seemed to be going round and the sky wheeling overhead. The sun was dodging about very strangely. Drops of sweat burst from the boy's forehead; he let fall his pole and said that he thought he would go home. The fellow who gave him the tobacco began to laugh and the other fellows to mock, but my boy did not mind them. Somehow, he did not know how he got out of the canal boat and started homeward, but at every step the ground rose as high as his knees before him, and then, when he got his foot high enough and began to put it down, the ground was not there. He was deathly sick, as he reeled and staggered on, and when he reached home and showed himself, white and haggard, to his frightened mother, he had scarcely strength enough to gasp out a confession of his attempt to retrieve the family honor by learning to chew tobacco. In another moment nature came to his relief, and then he fell into a deep sleep which lasted the whole afternoon, so that it seemed to him the next day when he woke up, glad to find himself alive if not so very lively.

Perhaps he had swallowed some of the poisonous juice of the tobacco; perhaps it had acted upon his brain without that. His father made no very close inquiry into the facts, and he did not forbid him the use of tobacco. It was not necessary; in that one little experiment he had got enough for a whole lifetime. It shows that after all a boy is not so hard to satisfy in everything.—*Harper's Young People.*

